# The Impact of Rhizospheric and Endophytic Bacteria on the Germination of *Carajasia cangae*: A Threatened Rubiaceae of the Amazon Cangas

**DOI:** 10.3390/microorganisms12091843

**Published:** 2024-09-06

**Authors:** Daniela Boanares, Aline Figueiredo Cardoso, Diego Fernando Escobar Escobar, Keila Jamille Alves Costa, José Augusto Bitencourt, Paulo Henrique O. Costa, Silvio Ramos, Markus Gastauer, Cecilio Frois Caldeira

**Affiliations:** Instituto Tecnológico Vale, Belém 66055-090, PA, Brazil; danielaboanares@gmail.com (D.B.); aline_f_cardoso@hotmail.com (A.F.C.); diego.escobar@pq.itv.org (D.F.E.E.); keila.costa@pq.itv.org (K.J.A.C.); jose.augusto.bitencourt@itv.org (J.A.B.); paulo.henrique.costa@pq.itv.org (P.H.O.C.); silvio.ramos@itv.org (S.R.); markus.gastauer@itv.org (M.G.)

**Keywords:** plant propagation, seeds, endemism, plant growth bacteria, campos rupestres

## Abstract

*Carajasia cangae* (Rubiaceae) is a narrow endemic species from the canga ecosystems of the Carajás National Forest that is facing extinction due to a limited range and habitat disturbance from hydroclimatological changes and mining activities. This study examines the influence of rhizospheric and endophytic bacteria on *C. cangae* seed germination to support conservation efforts. Soil samples, both rhizospheric and non-rhizospheric, as well as plant root tissues, were collected. Bacteria from these samples were subsequently isolated, cultured, and identified. DNA sequencing revealed the presence of 16 isolates (9 rhizospheric and 7 endophytic), representing 19 genera and 6 phyla: *Proteobacteria*, *Actinobacteria*, *Acidobacteria*, *Firmicutes*, *Bacteroidetes*, and *Chloroflexi*. The endophytic isolates of *Bacillus* and the rhizospheric isolates of *Planococcus* and *Lysinibacillus* reduced the median germination time and initiation time, while the rhizospheric isolates *Serratia* and *Comamonas* increased the germination time and decreased the germination percentage in comparison to the control sample. These findings emphasize the crucial role of endophytic bacteria in the germination of *C. cangae* and highlight isolates that could have beneficial effects in the following stages of plant growth. Understanding the impact of endophytic and rhizospheric bacterial isolates on seed germination can enhance conservation efforts by shortening the germination period of this species and thereby improving seedling production. Additionally, this knowledge will pave the way for future research on the role of bacteria in the establishment of *C. cangae*.

## 1. Introduction

Habitat loss and degradation disproportionately affect rare and endemic plant species, exacerbating the risk of extinction. These species often have specialized habitat requirements and limited geographic ranges, making them particularly vulnerable to habitat disturbances [1,2]. As their habitats shrink or become fragmented, rare and endemic plants face increased isolation, reduced population sizes, and heightened susceptibility to environmental pressures. Without targeted conservation measures, the loss of these unique species not only diminishes global plant diversity but also undermines the productivity, stability, and resilience of entire ecosystems [3].

The Amazon, recognized for its luxuriant forests, is also home to intricate and fragile ecosystems, like the campos rupestres over cangas or *canga* [2]. These mountainous regions with iron formations are covered by a rupestrian vegetation embedded within a forest matrix and are isolated based on altitude and substrate [4]. Despite their relatively small surface area, these environments are unique, harboring high levels of endemism and geographically restricted rare species [5,6,7,8]. Marked by a poorly developed, acidic, and iron-rich substrate and microclimatic diversity, these ecosystems selected for a highly specialized flora [9]. Nonetheless, as the Amazon forest has undergone continuous environmental degradation, markedly by land cover changes and pastureland expansions, local climate changes with increasing temperature and reduced water availability [10] add a new layer of pressure over these environmentally *restricted* species.

**Figure 1:**
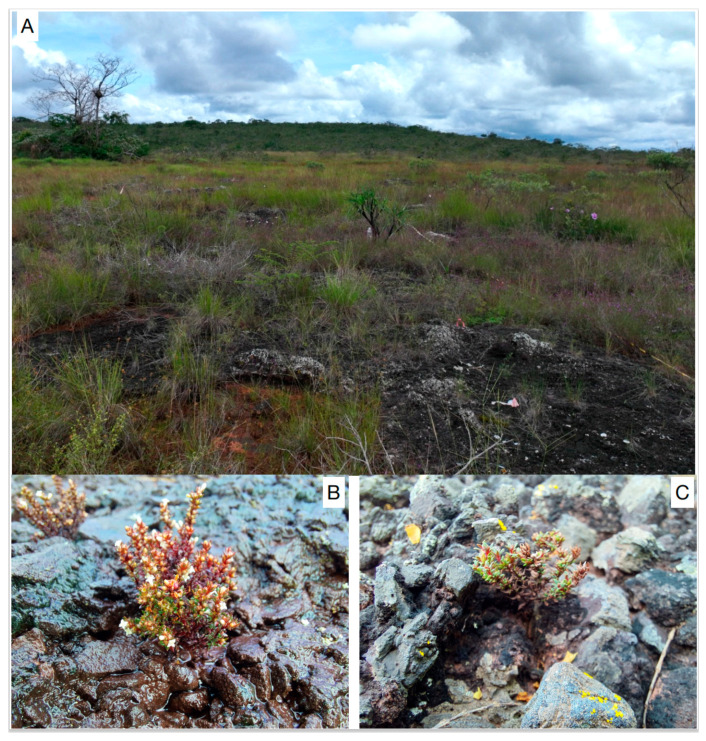
Images of the habitat (**A**) and plants of *Carajasia cangae* during the wet (**B**) and dry (**C**) seasons in *canga* ecosystems in Serra dos Carajás, eastern Amazon.

In the Serra dos Carajás (eastern Amazon), 38 plant species were identified and show edaphic endemism [5]. *Carajasia cangae* R.M. Salas, E. L. Cabral and Dessein (Rubiaceae), one of the most environmentally restrictive species, is a monotypic rupicolous herb recently described, growing 2 to 10 cm tall, perennial, with reddish leaves and stems, having insects as potential pollinators [11,12]. Considered highly restricted endemic, this species adapted to grow exclusively over ferruginous rocks (Figure 1) and has Area of Occupancy (AOO) and Extent of Occurrence (EOO) inferior to 50 km^2^ [5], coinciding with the extension of the *canga* plateau named S11, which harbors one of the world’s most important iron ore deposits [13]. In the canga environments, iron is present in minerals, often within chemical bonds, as found in magnetite (Fe^2+^ and Fe^3+^), hematite (Fe^3+^), and goethite (Fe^3+^) [14].The magnitude of its iron ore reserves and the current mining activities in the region also pose another significant threat to the species [4], already recognized as an endangered plant species [15].

Protecting endemic *canga* species requires a comprehensive understanding of their morphology, distribution, population dynamics, demography, genetics, propagation, adaptation, and habitat suitability, thereby informing conservation and management strategies [16,17,18,19]. In addition to traditional conservation methods, additional strategies may be necessary to ensure preservation, including the establishment of ex situ cultivation, germplasm bank formation, and the reintroduction or reinforcement of extant populations. Enhancing survival rates could involve measures such as improving seed germination to facilitate species propagation, growth, and successful establishment. This could be particularly vital for *C. cangae* due to its small population size, specific habitat requirements, and reliance on environmental stability, which increases its vulnerability [20].

Seed manipulation represents a viable and recommended strategy for enhancing the survival of rare or endemic plant species [21], such as *C. cangae*. These species often rely on intricate interactions to thrive in their habitat, including symbiotic relationships during germination. Understanding these complex interactions is pivotal for conservation efforts aimed at protecting them. In the case of *C. cangae*, factors such as limited seed availability, seed dormancy, and a low germination percentage [22] may pose significant challenges to seedling establishment and growth. Plant growth-promoting bacteria (PGPB) are well documented for their beneficial effects on plant physiology and metabolism, including hormone regulation, nutrient absorption facilitation, and enhancement of resistance to biotic and abiotic stresses [23]. However, the influence of PGPB on seed germination can vary among species, with effects ranging from positive to negative or neutral [24,25,26,27,28]. Our research aims to investigate the effects of bacteria on *C. cangae* seed germination, focusing on both endophytic and rhizospheric bacteria. These bacteria, isolated from the rhizosphere and root system of *C. cangae* in its natural habitat, provide valuable insights into seed germination, a key factor for enhancing propagation and conservation efforts.

## 2. Materials and Methods

### 2.1. Study Area and Sample Collection

The study was conducted in the Carajás National Forest, located in Serra dos Carajás, Eastern Amazon, Pará, Brazil. The forest lies between latitudes 5°52′ and 6°32′ S and longitudes 49°53′ and 50°49′ W, experiencing a hot tropical climate classified as Aw by the Köppen system. With an annual precipitation of approximately 2000 mm, rainfall is concentrated from November to March during the rainy season, while a dry period spans May to September [29]. The samples were collected in the rainy season of 2023.

Nine field sampling trips were undertaken to collect *C. cangae* plants and substrate from non-rhizospheric areas (those outside the immediate influence of plant roots) and rhizospheric areas (those adjacent to the roots), collected by using sterile spatulas and tweezers. The roots of two plants were sampled for microbial isolation, and soil samples were individually collected near the roots by using sterile spatulas.

### 2.2. Isolation of Rhizospheric Bacteria

Rhizospheric samples from *C. cangae* were collected, with 2 g of each sample being diluted in 25 mL of sterile distilled water and agitated for 30 min. A 20 μL aliquot was then taken from the suspension and further diluted in 80 μL of distilled water. Subsequently, 50 μL aliquots were added to triplicate Petri dishes, each containing sterile Kado and Heskett culture medium 523, a basic medium that has sucrose as its carbon source [30]. Kado and Heskett culture medium 523 is a non-selective medium formulated with essential nutrients to support basic microbial growth. Our objective was to cultivate a diverse range of colonies, increasing the chances of isolating strains with growth-promoting properties. 

Incubation was carried out at 27 °C with a 12 h photoperiod in a growth chamber (Incubator Shaker Series, Innova 42, Eppendorf, Hamburg, Germany). After incubation, colonies with distinct colors, edges, and morphology were isolated, purified, and stored in 15% glycerol at −80 °C for further processing [31]. 

### 2.3. Isolation and Suspension of Endophytic Bacteria

Root segments were superficially sterilized by performing sequential washing in 70% ethanol for 30 s and 2% sodium hypochlorite for one minute, and two washes with sterile distilled water. Following sterilization, root segments were macerated in 5 mL Eppendorf tubes containing 2 mL of sterile PBS buffer [32]. Subsequently, 50 μL aliquots were added to triplicate Petri dishes, each containing sterile Kado and Heskett culture medium 523 [30], followed by incubation in a growth chamber at 27 °C with a 12 h photoperiod. After incubation, colonies with distinct characteristics were isolated, purified, and stored in 15% glycerol at −80 °C. The bacterial suspension was prepared according to Filippi et al. (2011), with each isolated bacterium being grown in liquid medium [30] under agitation at 114 rpm at 28 °C for 24 h, and the concentration was adjusted to 10^8^ CFU⋅mL^–1^ [33].

### 2.4. DNA Extraction and Sequencing

The DNA extraction and sequencing of individual isolates and soil and rhizospheric samples were performed to analyze the structure of bacterial communities (3 reps for each condition). The DNA was extracted from 250 mg of soil by using the QIAGEN PowerSoil Pro^®^ DNA Isolation Kit (QIAGEN, Hilden, Germany), following the manufacturer’s instructions [34]. The DNA concentration was measured by using the Qubit^®^ 3.0 (Thermo Fisher Scientific, Waltham, MA, USA). DNA quality was assessed by using 1% agarose gel electrophoresis (Life Technologies, Thermo Fisher Scientific Inc., Waltham, MA, USA). The construction of bacterial sequence libraries was performed by using the Illumina 16S Metagenomic Sequencing Library Preparation protocol (Illumina, San Diego, CA, USA). Bacterial community analyses were conducted on the V3–V4 regions of the 16S rRNA gene, which were amplified by polymerase chain reaction (PCR). The primer pair “S-D-Bact-0341-b-S-17-N” (5′-TCGTCGGCAGCGTCAGATGTGTATAAGAGACAGCCTACGGGNGGCWGCAG-3′) and “S-D-Bact-0785-a-A-21-N” (5′-GTCTCGTGGGCTCGGAGATGTGTATAAGAGACAGGACTACHVGGGTATCTAATCC-3′) with adaptors for the MiSeq-Illumina platform (Illumina, San Diego, CA, USA) was used. The bacterial primers contained nucleotide sequences called overhangs, which allowed for the addition of indexes in subsequent steps. Negative controls were included during DNA extraction and PCR.

For the first PCR, 12.5 µL of 2x Kapa HiFi HotStart Ready Mix enzyme, 5 µL of each primer, and 2.5 µL of DNA were used. The library construction for bacterial fragments included an initial step in the thermocycler at 95 °C for 3 min, followed by 25 cycles at 95 °C for 30 s, 55 °C for 30 s, 72 °C for 30 s, and a final extension step at 72 °C for 5 min. DNA quantification was then performed by using the Qubit™ dsDNA HS (High Sensitivity) Assay Kit (Thermo Fisher Scientific, Waltham, MA, USA)and the Qubit^®^ 3.0 fluorometer (Thermo Fisher Scientific, Waltham, MA, USA). Amplicon quality, regarding fragment size, was assessed by using capillary electrophoresis with the Agilent Technology 2100 Bioanalyzer. The PCR products were purified by using AMPure XP magnetic beads (Beckman Coulter, Brea, CA, USA) and indexed in a new PCR with the Nextera XT kit (Illumina, San Diego, CA, USA), where unique sequence adapters (indexes) were added to each sample. For the second PCR, 2.5 µL of Nextera XT index Primer1, 2.5 µL of Nextera XT index Primer2, 12.5 µL of 2x Kapa HiFi HotStart Ready Mix enzyme, 5 µL of ultrapure water, and 2.5 µL of PCR product were used. Subsequently, purification was performed again by using magnetic beads with the Agencourt AMPure XP kit (Beckman Coulter, Brea, CA, USA), and the library was quantified by using the Qubit^®^ 3.0 (Thermo Fisher Scientific, Waltham, MA, USA).

Next, the library was normalized to a concentration of 24 nM, and the genomic pool was prepared according to the Illumina 16S Metagenomic Sequencing Library Preparation protocol (Illumina, San Diego, CA, USA). Following library preparation, amplicon sequencing was performed on the MiSeq-Illumina platform by using the MiSeq V3 600-cycle sequencing kit (Illumina, San Diego, CA, USA).

### 2.5. Bioinformatics

The sequences obtained from the amplicon sequencing of the soil samples’ 16S regions were aligned by using the PIMBA (a Pipeline for MetaBarcoding Analysis), a pipeline based on QIIME (Quantitative Insights Into Microbial Ecology) [35,36,37]. The bacterial sequences underwent trimming steps to remove contaminants and adaptors. Quality filtering (Phred > 20) was performed by using the Prinseq tool. Subsequently, only the forward and reverse sequences were assembled by using the Pear assembler [38]. After assembly, dereplication was conducted, followed by truncation at 240 base pairs. Sequences shorter than 50 base pairs were discarded, and chimera filtering was performed. The sequences were clustered into Operational Taxonomic Units (OTUs) by using VSEARCH, and OTU clustering was used to classify groups of individuals with >97% similarity. Taxonomic classification was performed by using the Ribosomal Database Project (http://rdp.cme.msu.edu/, accessed on 20 January 2024).

### 2.6. Selection and Inoculation of Isolates and Germination Test

The experimental design consisted of 17 treatments (16 rhizobacterial and endophytic isolates and 1 control treatment—T0 containing Kado and Heskett culture medium 523 only) with 6 replicates each, containing 20 seeds per Petri dish, for a total of 2.040 seeds. *Carajasia cangae* seeds were harvested from *canga* body S11B, Serra dos Carajás, Brazil. The seeds were sterilized with 70% ethanol and 4% sodium hypochlorite for 1 min in each solution, and they were washed 3 times with distilled water. In each treatment, i.e., rhizobacteria isolate, 20 seeds were submerged in a 2 mL bacterial suspension of 10^8^ CFU·mL^–1^ for five minutes. After this period, excess liquid was removed, and the seeds were sown on sterilized filter paper in Petri dishes that had been previously autoclaved for 1 h at 121 °C. The Petri dish was constantly wetted with distilled water.

For the germination test, the Petri dishes containing the seeds were kept in plant growth chambers (Fitotron SGC120; Weiss Technik, United Kingdom) at constant temperature of 25 °C under an irradiance of 50 μmol m^−2^ s^−1^ with a 12 h light/dark photoperiod. Evaluation was conducted every seven days for two months. Seeds were monitored and considered germinated when radicle protrusion (2 mm) was observed.

### 2.7. Data Analysis

The statistical analyses were carried out in the R environment by using the packages ggplot v.3.3.0, vegan v.2.5-6, and phyloseq v.1.30.0 [39]. Taxonomic composition was used to construct graphs for each soil sample, considering relative abundance matrices. To evaluate the relationship between different isolates and *C. cangae* germination, the treatments were compared by using time-to-event analysis. In this type of analysis, germination curves were constructed for each treatment by using a parametric model, specifically the log-linear model. Subsequently, a grouped-permutation likelihood ratio test was conducted to determine if the germination curves differed between treatments. The grouped-permutation test considers that the seeds are grouped in Petri dishes. The germination percentage, median germination time (T50), and time to start seed germination (T10) of the treated seeds were compared with control seed by means of the comPar function in the drc package in R [40].

## 3. Results

### 3.1. Soil Bacterial Communities

**Figure 2:**
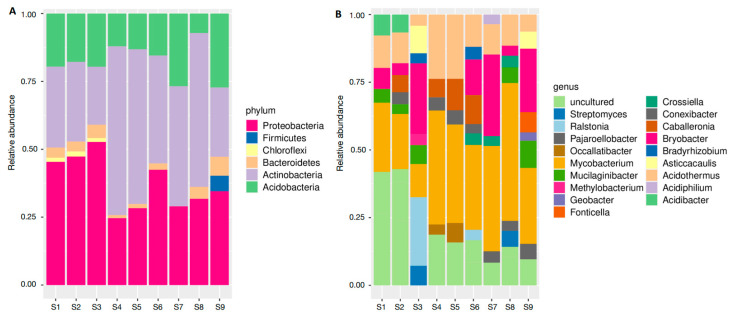
Identification of the communities of bacterial 16S rRNA obtained from soil samples nearby *Carajasia cangae* plants growing in a *canga* ecosystem in Serra dos Carajás, eastern Amazon. The relative abundance of the (**A**) phyla and detailed by (**B**) genus.

A total of 18 isolates distributed across 6 phyla and 18 genera were identified in the collected soil samples. Considering only the sequences with some level of taxonomic identification, Proteobacteria (41.3% of all OTUs), Actinobacteria (40.3%), Acidobacteria (17.4%), Firmicutes (5.7%), Bacteroidetes (4%), and Chloroflexi (1.6%) were the phyla found (Figure 2A). Among them, the most representative genera were *Mycobacterium* (34%), *Acidobacter* (19%), and *Acidothermus* (15%) (Figure 2B; Supplemental Table S1).

### 3.2. Rhizospheric and Endophytic Isolates Isolation and Molecular Identification

**Figure 3:**
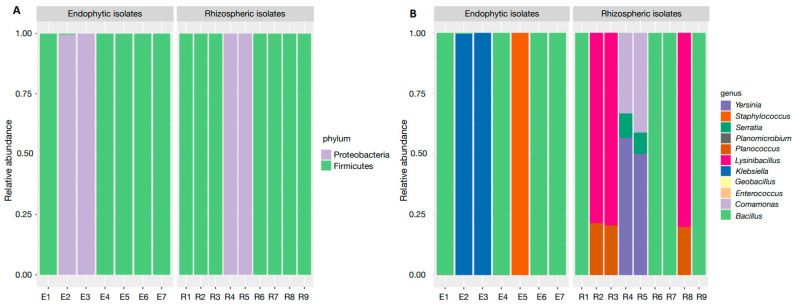
Identification of the communities of bacterial 16S rRNA obtained from *Carajasia cangae* rhizosphere and roots from plants growing in a *canga* ecosystem in Serra dos Carajás, eastern Amazon. The relative abundance of the (**A**) phyla and detailed by (**B**) genus.

We obtained bacterial isolates from plant samples, i.e., endophytic, and rhizospheric soil of *C. cangae*. Sixteen bacterial isolates were obtained, of which nine were rhizospheric and seven were endophytic (Figure 3 and Supplemental Table S2). From these 16 isolates isolated (rhizospheric soil and roots of *C. cangae*), a total of 131 OTUs of different bacteria were detected. Some samples were made up of more than one isolate. Considering only the sequences with some level of taxonomic identification, the isolates were present in only two phyla: Firmicutes and Proteobacteria. Among the 11 identified genera, *Bacillus* (47%), *Comamonas* (27%) and *Serratia* (21%) were the most abundant. Among the identified species are *Bacillus cereus*, *B. subtilis*, *Lysinobacillus fusiformis*, *Serratia marcescens*, and *Klebsiella pneumoniae* (Figure 3).

### 3.3. Seed Germination

The germination curves differed significantly according to the germination treatments (Likelihood Ratio—LR = 708.6; *p*-value = 0.005). Regarding the germination percentage, no isolates had a positive interference compared with the control. Among the endophytic isolates, E2 (*Klebsiella*), E4 (*Bacillus*), and E5 (*Staphylococcus*) had a negative effect on the germination percentage of *C. cangae* seeds. E6 (*Bacillus*) reduced the time for half of the seeds to germinate.

**Figure 4:**
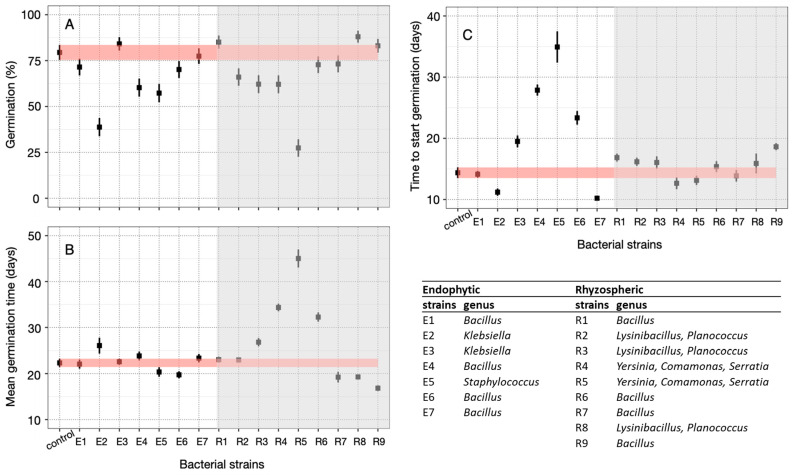
Germinability of *Carajasia cangae* seeds treated with 16 different bacterial isolates. Germination (**A**), median germination time (days) (**B**), and time to start germination (days) (**C**). The germination test was carried out for 60 days under the constant temperature of 25 °C under an irradiance of 50 μmol m^−2^ s^−1^ with a 12 h light/dark photoperiod. Error bars represent the standard deviation at 95%. Red horizontal lines represent the error bars of the control treatment.

Regarding the rhizospheric isolates, R2-R3 (*Planococcus* and *Lysinibacillus*) and R4-R5 *(Comamonas*, *Serratia*, and *Yersinia*), showed a negative effect on the germination percentage (Figure 4A). On the other hand, isolates R7 and R9 (*Bacillus*) reduced the median germination time, while isolates R1 and R6 (*Bacillus*), R2 and R3 (*Planococcus* and *Lysinibacillus*), and R4 and R5 (*Comamonas*, *Serratia*, and *Yersinia)* delayed the onset of germination (Figure 4B). Isolates R7, R9, and R8 (*Planococcus* and *Lysinibacillus*), reduced the time for half of the seeds to germinate by 3 to 6 days (Figure 4C). Conversely, isolates R3 (*Planococcus* and *Lysinibacillus*), R4 and R5 (*Comamonas*, *Serratia*, and *Yersinia*), and R6 (*Bacillus*) negatively influenced this parameter.

## 4. Discussion

Although the role of plant growth-promoting bacteria (PGPB) in the growth and establishment of various plant species is well documented, this study represents the first examination of their impact on germination within Amazonian *canga* ecosystems. Germination is a critical bottleneck that must be addressed when considering strategies for translocation, reintroduction, enhancing gene flow, and promoting establishment, as well as for conservation efforts. In situ and ex situ conservation strategies each have their advantages and drawbacks. In situ conservation focuses on maintaining natural processes and interactions vital for species survival, but it may not effectively tackle certain issues, like seed germination and seedling establishment. On the other hand, ex situ methods, such as seed banks, tissue culture, and botanical gardens, play a critical role in preserving genetic diversity away from natural habitats. However, while these ex situ approaches safeguard genetic material, they do not always guarantee successful reintroduction into natural environments. Understanding the specific role of each bacterial isolate in germination is essential to optimizing success in the seed germination process. However, it is important to note that isolates may initially exhibit either beneficial or inhibitory effects on germination but can have different impacts along the plant life cycle. 

In our study, we observed that none of the bacterial isolates identified in the rhizosphere or endophytes were present in the soil samples taken just a few centimeters away from the plants. This occurrence may be attributed to habitat selectivity. *Carajasia cangae* predominantly thrives in exposed iron rock fields, an environment to which very few species are adapted, where conventional soil is absent. As a result, soil samples collected near these rocks’ bases may show significant differences in microbial populations compared with this unique microhabitat. This environment requires specific adaptations for plant survival, including those related to high temperatures, irradiation, and water scarcity. Such habitat selectivity may not only influence plant distribution but also microbial communities.

Strains isolated from the plant or its surroundings may exhibit similar effects on germination, indicating that their influence could be independent of their isolation source. Among the most abundant bacteria, the genus *Bacillus*, found in most of the samples from the rhizosphere and plants, played a significant role in reducing the germination time of *C. cangae* in relation to the control, corroborating the fact that *Bacillus* are among the most abundant soil residents with various PGPB characteristics, adding this function of improving the germination rate [41]. These bacteria use several mechanisms to enhance plant growth and productivity through direct and indirect influence under various environmental conditions [42]. However, some isolates identified as *Bacillus* negatively interfered with the germination process. One reason could be the fact that *Bacillus* sp. can produce hydrogen cyanide gas, which becomes toxic to seeds in large quantities and inhibits germination [43,44].

Among the identified isolates are *Bacillus subtilis* and *B. cereus*. The role of *B. subtilis* in improving the germination of some species is already known [45]. Like some PGPB, *B. subtilis* and *B. cereus* can synthesize plant hormones through their secondary metabolism, such as auxin (AIA, indole acetic acid) and gibberellin (GA) [46]. The biosynthesis of gibberellic acid can promote germination by triggering the activities of α-amylase [47]. The improved seed germination can also be attributed to a solution retention effect with the isolates in the pre-germination phase, which subsequently influences vital metabolic processes [48]. A similar effect was observed in *Bacillus tropicus*, which enhanced the germination of wheat seeds [49]. In addition to *B. subtilis*, *Planococcus* sp. also positively influenced the germination of *C. cangae*. Both bacteria species are known to produce ACC deaminase [50,51]. This enzyme plays a vital role in plant–ethylene interactions by catalyzing the conversion of ACC, the immediate precursor of ethylene biosynthesis in higher plants, into α-ketobutyrate and ammonia. Consequently, bacteria can utilize the ammonia released from ACC as a nitrogen source, effectively reducing the ethylene levels in the plant.

As observed for *Planococcus sp.*, *Lysinobacillus fusiformis* also improved the germination rate of *C. cangae* and exhibited similar activities [51,52]. These bacteria can produce enzymes such as protease, urease, catalase, cellulase, and amylase, which are related to processes underlying seed germination [53,54]. Catalase promotes the balance between ROS production and elimination, which must be under elaborate and rigorous control for seed germination [55,56,57].

Strains of *Klebsiella pneumoniae* have been described as plant growth promoters [58,59] because of their ability to produce auxin. However, in this study, we observed a reduction in the seed germination percentage of *C. cangae*. Such results may be linked to the non-beneficial effect of IAA on seed germination; as observed in soybean, exogenous auxin application can suppress seed germination by mediating ABA and GA biosynthesis, delaying seed coat rupture and radicle protrusion [60]. Conversely, in *Salicornia bigelovii*, *Klebsiella pneumoniae* enhanced seed germination even under high-salinity conditions, while *Klebsiella aerogenes* increased the relative germination rate in carrot seeds [61,62]. 

*Serratia marcescens* and *Comamonas* sp., identified on rhizospheric samples, drastically limited the germination of *C. cangae*. Although these bacteria have some isolates related to PGPB, very few *Comamonas* spp. have been found to be responsible for plant growth-promoting activities [63]. It is possible that these bacteria did not improve the germination of *C. cangae*, but they still play a significant role in plant growth. It is important to highlight that the isolates that positively affected *C. cangae* germination were found in the rhizosphere and within the roots, both intercellularly and intracellularly. The interior of the plant is colonized by a variety of endophytes derived mainly from the rhizosphere, and many of them have been reported to improve plant growth [64]. Bacteria such as *Yersinia*, also found together with *Comamonas* and *Serratia* isolates, may be important for phosphate solubilization in the plant, with no other activities favoring seed germination [65]. Certain *Bacillus* species, including *B. subtilis*, *B. megaterium*, and *B. simplex*, also exhibit this characteristic [66,67,68].

The availability of nutrients is crucial for species establishment, especially in environments like the *C. cangae* habitat, which is known for its nutritional restrictions. Although our evaluation focused solely on germination, subsequent stages, particularly seedling establishment, heavily rely on nutrient availability, with phosphorus being particularly essential but typically limited. From the isolates we observed in this study, we can also expect a benefit effect on stages following germination, as such isolates can play a more significant role in seedling growth and establishment rather than germination itself. This may explain why we did not observe significant benefits during the initial germination process for some of them, as seen in *Bacillus* sp. Our research indicates that applying PGPB can enhance seed germination and, potentially, seedling growth, thereby improving the effectiveness of both in situ and ex situ conservation efforts. Recognizing the pivotal role of bacterial communities in the rhizosphere for plant growth and development, PGPB have the potential to facilitate successful colonization in other areas for reintroduction purposes. This is especially pertinent when considering the reintroduction of endangered species. PGPB, by directly or indirectly shielding plants from biotic and abiotic stresses, could be instrumental in the establishment of species, particularly in challenging environments during their reintroduction.

## 5. Conclusions

*Carajasia cangae* may benefit from associating its germination with plant growth-promoting bacteria (PGPB). *Bacillus* sp. and the combination of *Planococcus* and *Lysinibacillus* positively affected the germination of *C. cangae*, while some isolates showed negative effects. However, these isolates possess characteristics beneficial for subsequent stages of seedling development and establishment, such as siderophore production and nutrient availability (e.g., *Yersinia* sp.). Understanding the germination behavior of rare and endemic species like *C. cangae* is crucial for effective conservation and management strategies. Ecological methods involving endophytic and rhizospheric bacteria can aid in the propagation and preservation of such species. Together with PGPB, in this study, we identified a diverse range of bacterial taxa, providing insights into soil biodiversity in the study area. Further research and conservation efforts are essential to ensuring the survival of unique species like *C. cangae* and protect the biodiversity of threatened Amazonian ecosystems.

## Data Availability

The datasets used and/or analyzed during the current study are available from the corresponding author upon reasonable request.

## Supplementary Materials

The following supporting information can be downloaded at: https://www.mdpi.com/article/10.3390/microorganisms12091843/s1, Table S1: Most abundant bacterial taxa identified in soils associated with Carajasia cangae.; Table S2: Most abundant bacterial taxa identified and bacterial 16S rRNA sequences in different samples associated with Carajasia cangae growing in a canga in Serra dos Carajás, Eastern Amazon.

## References

[1] Devecchi M.F., Lovo J., Moro M.F., Andrino C.O., Barbosa-Silva R.G., Viana P.L., Giulietti A.M., Antar G., Watanabe M.T.C., Zappi D.C. (2020). Beyond Forests in the Amazon: Biogeography and Floristic Relationships of the Amazonian Savannas. Bot. J. Linn. Soc..

[2] Zappi D.C., Moro M.F., Walker B., Meagher T., Viana P.L., Mota N.F.O., Watanabe M.T.C., Nic Lughadha E. (2019). Plotting a Future for Amazonian Canga Vegetation in a Campo Rupestre Context. PLoS ONE.

[3] Petchey O.L. (2000). Species Diversity, Species Extinction, and Ecosystem Function. Am. Nat..

[4] Mota N.F.D.O., Watanabe M.T.C., Zappi D.C., Hiura A.L., Pallos J., Viveros R.S., Giulietti A.M., Viana P.L. (2018). Amazon Canga: The Unique Vegetation of Carajás Revealed by the List of Seed Plants. Rodriguésia.

[5] Giulietti A.M., Giannini T.C., Mota N.F.O., Watanabe M.T.C., Viana P.L., Pastore M., Silva U.C.S., Siqueira M.F., Pirani J.R., Lima H.C. (2019). Edaphic Endemism in the Amazon: Vascular Plants of the Canga of Carajás, Brazil. Bot. Rev..

[6] Jacobi C.M., Do Carmo F.F., Vincent R.C., Stehmann J.R. (2007). Plant Communities on Ironstone Outcrops: A Diverse and Endangered Brazilian Ecosystem. Biodivers. Conserv..

[7] Viana P.L., Mota N.F.D.O., Gil A.D.S.B., Salino A., Zappi D.C., Harley R.M., Ilkiu-Borges A.L., Secco R.D.S., Almeida T.E., Watanabe M.T.C. (2016). Flora Das Cangas Da Serra Dos Carajás, Pará, Brasil: História, Área de Estudos e Metodologia. Rodriguésia.

[8] Zappi D.C., Moro M.F., Meagher T.R., Nic Lughadha E. (2017). Plant Biodiversity Drivers in Brazilian Campos Rupestres: Insights from Phylogenetic Structure. Front. Plant Sci..

[9] Skirycz A., Castilho A., Chaparro C., Carvalho N., Tzotzos G., Siqueira J.O. (2014). Canga Biodiversity, a Matter of Mining. Front. Plant Sci..

[10] Souza-Filho P.W.M., De Souza E.B., Silva Júnior R.O., Nascimento W.R., Versiani De Mendonça B.R., Guimarães J.T.F., Dall’Agnol R., Siqueira J.O. (2016). Four Decades of Land-Cover, Land-Use and Hydroclimatology Changes in the Itacaiúnas River Watershed, Southeastern Amazon. J. Environ. Manag..

[11] Salas R.M., Viana P.L., Cabral E.L., Dessein S., Janssens S. (2015). Carajasia (Rubiaceae), a New and Endangered Genus from CarajáS Mountain Range, Pará Brazil. Phytotaxa.

[12] Pinto C.E., Awade M., Watanabe M.T.C., Brito R.M., Costa W.F., Maia U.M., Imperatriz-Fonseca V.L., Giannini T.C. (2020). Size and Isolation of Naturally Isolated Habitats Do Not Affect Plant-Bee Interactions: A Case Study of Ferruginous Outcrops within the Eastern Amazon Forest. PLoS ONE.

[13] Lindenmayer Z.G., Laux J.H., Teixeira J.B.G. (2001). Considerações Sobre a Origem Das Formações Ferríferas Da Formação Carajás, Serra Dos Carajás. RBG.

[14] Spier C.A., Levett A., Rosière C.A. (2019). Geochemistry of Canga (Ferricrete) and Evolution of the Weathering Profile Developed on Itabirite and Iron Ore in the Quadrilátero Ferrífero, Minas Gerais, Brazil. Min. Depos..

[15] Amorim E., Bicalho M. (2022). Carajasia cangae (Rubiaceae).

[16] Caldeira C.F., Abranches C.B., Gastauer M., Ramos S.J., Guimarães J.T.F., Pereira J.B.S., Siqueira J.O. (2019). Sporeling Regeneration and Ex Situ Growth of Isoëtes Cangae (Isoetaceae): Initial Steps towards the Conservation of a Rare Amazonian Quillwort. Aquat. Bot..

[17] Dalapicolla J., Alves R., Jaffé R., Vasconcelos S., Pires E.S., Nunes G.L., Pereira J.B.D.S., Guimarães J.T.F., Dias M.C., Fernandes T.N. (2021). Conservation Implications of Genetic Structure in the Narrowest Endemic Quillwort from the Eastern Amazon. Ecol. Evol..

[18] Monteiro W.P., Dalapicolla J., Carvalho C.S., Costa Veiga J., Vasconcelos S., Ramos S.J., Gastauer M., Jaffé R., Caldeira C.F. (2022). Genetic Diversity and Structure of an Endangered Medicinal Plant Species (*Pilocarpus microphyllus*) in Eastern Amazon: Implications for Conservation. Conserv. Genet..

[19] Caldeira C.F., Giannini T.C., Ramos S.J., Vasconcelos S., Mitre S.K., Pires J.P.D.A., Ferreira G.C., Ohashi S., Mota J.A., Castilho A. (2017). Sustainability of Jaborandi in the Eastern Brazilian Amazon. Perspect. Ecol. Conserv..

[20] Zappi D.C., Miguel L.M., Sobrado S.V., Salas R.M. (2017). Flora Das Cangas Da Serra Dos Carajás, Pará, Brasil: Rubiaceae. Rodriguésia.

[21] Bairu M.W., Kulkarni M.G., Street R.A., Mulaudzi R.B., Van Staden J. (2009). Studies on Seed Germination, Seedling Growth, and In Vitro Shoot Induction of Aloe Ferox Mill., a Commercially Important Species. horts.

[22] Zanetti M., Dayrell R.L.C., Wardil M.V., Damasceno A., Fernandes T., Castilho A., Santos F.M.G., Silveira F.A.O. (2020). Seed Functional Traits Provide Support for Ecological Restoration and Ex Situ Conservation in the Threatened Amazon Ironstone Outcrop Flora. Front. Plant Sci..

[23] Majeed S., Nawaz F., Naeem M., Ashraf M.Y. (2018). Effect of Exogenous Nitric Oxide on Sulfur and Nitrate Assimilation Pathway Enzymes in Maize (*Zea mays* L.) under Drought Stress. Acta Physiol. Plant.

[24] Dutta S., Sarkar A., Dutta S. (2019). Characterization of Pseudomonas Aeruginosa MCC 3198 and Its Potential for Growth Promotion of Seedlings of the Medicinal Plant *Celosia cristata* L. Int. J. Curr. Microbiol. App. Sci.

[25] Pavlova A.S., Leontieva M.R., Smirnova T.A., Kolomeitseva G.L., Netrusov A.I., Tsavkelova E.A. (2017). Colonization Strategy of the Endophytic Plant Growth-Promoting Strains of *Pseudomonas fluorescens* and *Klebsiella oxytoca* on the Seeds, Seedlings and Roots of the Epiphytic Orchid, *Dendrobium nobile* Lindl. J. Appl. Microbiol..

[26] Tsavkelova E.A., Egorova M.A., Leontieva M.R., Malakho S.G., Kolomeitseva G.L., Netrusov A.I. (2016). Dendrobium Nobile Lindl. Seed Germination in Co-Cultures with Diverse Associated Bacteria. Plant Growth Regul..

[27] Tsavkelova E.A., Cherdyntseva T.A., Klimova S.Y., Shestakov A.I., Botina S.G., Netrusov A.I. (2007). Orchid-Associated Bacteria Produce Indole-3-Acetic Acid, Promote Seed Germination, and Increase Their Microbial Yield in Response to Exogenous Auxin. Arch. Microbiol..

[28] Zhao K., Li J., Zhang X., Chen Q., Liu M., Ao X., Gu Y., Liao D., Xu K., Ma M. (2018). Actinobacteria Associated with Glycyrrhiza Inflata Bat. Are Diverse and Have Plant Growth Promoting and Antimicrobial Activity. Sci. Rep..

[29] Alvares C.A., Stape J.L., Sentelhas P.C., De Moraes Gonçalves J.L., Sparovek G. (2013). Köppen’s Climate Classification Map for Brazil. metz.

[30] Kado C., Heskett M. (1970). Selective Media for Isolation of Agrobacterium, Corynebacterium, Erwinia, Pseudomonas and Xanthomonas. Phytopathology.

[31] Bircher L., Schwab C., Geirnaert A., Lacroix C. (2018). Cryopreservation of Artificial Gut Microbiota Produced with In Vitro Fermentation Technology. Microb. Biotechnol..

[32] Mendes R., Pizzirani-Kleiner A.A., Araujo W.L., Raaijmakers J.M. (2007). Diversity of Cultivated Endophytic Bacteria from Sugarcane: Genetic and Biochemical Characterization of *Burkholderia cepacia* Complex Isolates. Appl. Environ. Microbiol..

[33] Filippi M.C.C., Da Silva G.B., Silva-Lobo V.L., Côrtes M.V.C.B., Moraes A.J.G., Prabhu A.S. (2011). Leaf Blast (*Magnaporthe oryzae*) Suppression and Growth Promotion by Rhizobacteria on Aerobic Rice in Brazil. Biol. Control.

[34] Costa P.H.D.O., Nascimento S.V.D., Herrera H., Gastauer M., Ramos S.J., Caldeira C.F., Oliveira G., Valadares R.B.D.S. (2021). Non-Specific Interactions of Rhizospheric Microbial Communities Support the Establishment of *Mimosa acutistipula* Var. Ferrea in an Amazon Rehabilitating Mineland. Processes.

[35] Bolyen E., Rideout J.R., Dillon M.R., Bokulich N.A., Abnet C.C., Al-Ghalith G.A., Alexander H., Alm E.J., Arumugam M., Asnicar F. (2019). Reproducible, Interactive, Scalable and Extensible Microbiome Data Science Using QIIME 2. Nat. Biotechnol..

[36] Caporaso J.G., Kuczynski J., Stombaugh J., Bittinger K., Bushman F.D., Costello E.K., Fierer N., Peña A.G., Goodrich J.K., Gordon J.I. (2010). QIIME Allows Analysis of High-Throughput Community Sequencing Data. Nat. Methods.

[37] Oliveira R.R.M., Silva R., Nunes G.L., Oliveira G., Stadler P.F., Walter M.E.M.T., Hernandez-Rosales M., Brigido M.M. (2021). PIMBA: A PIpeline for MetaBarcoding Analysis. Advances in Bioinformatics and Computational Biology.

[38] Zhang J., Kobert K., Flouri T., Stamatakis A. (2014). PEAR: A Fast and Accurate Illumina Paired-End ReAd MergeR. Bioinformatics.

[39] McMurdie P.J., Holmes S. (2013). Phyloseq: An R Package for Reproducible Interactive Analysis and Graphics of Microbiome Census Data. PLoS ONE.

[40] Ritz C., Baty F., Streibig J.C., Gerhard D. (2015). Dose-Response Analysis Using R. PLoS ONE.

[41] Radhakrishnan R., Hashem A., Abd_Allah E.F. (2017). Bacillus: A Biological Tool for Crop Improvement through Bio-Molecular Changes in Adverse Environments. Front. Physiol..

[42] Hashem A., Tabassum B., Fathi Abd_Allah E. (2019). Bacillus Subtilis: A Plant-Growth Promoting Rhizobacterium That Also Impacts Biotic Stress. Saudi J. Biol. Sci..

[43] Tiwari S., Prasad V., Lata C. (2019). Bacillus: Plant Growth Promoting Bacteria for Sustainable Agriculture and Environment. New and Future Developments in Microbial Biotechnology and Bioengineering.

[44] Walia A., Mehta P., Chauhan A., Shirkot C.K. (2014). Effect of Bacillus Subtilis Strain CKT1 as Inoculum on Growth of Tomato Seedlings Under Net House Conditions. Proc. Natl. Acad. Sci. India Sect. B Biol. Sci..

[45] Ha-Tran D.M., Nguyen T.T.M., Hung S.-H., Huang E., Huang C.-C. (2021). Roles of Plant Growth-Promoting Rhizobacteria (PGPR) in Stimulating Salinity Stress Defense in Plants: A Review. IJMS.

[46] Tsukanova K.A., Chebotar V.K., Meyer J.J.M., Bibikova T.N. (2017). Effect of Plant Growth-Promoting Rhizobacteria on Plant Hormone Homeostasis. S. Afr. J. Bot..

[47] Liu J., Li L., Yuan F., Chen M. (2019). Exogenous Salicylic Acid Improves the Germination of *Limonium bicolor* Seeds under Salt Stress. Plant Signal. Behav..

[48] Zulfiqar F. (2021). Effect of Seed Priming on Horticultural Crops. Sci. Hortic..

[49] Bourak K., Oulkhir F.E., Maghnia F.Z., Massart S., Biskri L., Jijakli M.H., Allaoui A. (2024). A Comprehensive Approach Combining Short-Chain Polyphosphate and Bacterial Biostimulants for Effective Nutrient Solubilization and Enhanced Wheat Growth. Microorganisms.

[50] Mukhtar T., Rehman S.U., Smith D., Sultan T., Seleiman M.F., Alsadon A.A., Ali S.A., Chaudhary H.J., Solieman T.H.I., Ibrahim A.A. (2020). Mitigation of Heat Stress in *Solanum lycopersicum* L. by ACC-Deaminase and Exopolysaccharide Producing *Bacillus cereus*: Effects on Biochemical Profiling. Sustainability.

[51] Verma P., Yadav A.N., Khannam K.S., Kumar S., Saxena A.K., Suman A. (2016). Molecular Diversity and Multifarious Plant Growth Promoting Attributes of Bacilli Associated with Wheat (*Triticum aestivum* L.) Rhizosphere from Six Diverse Agro-Ecological Zones of India: Diversity and Plant Growth Promoting Attributes of Bacilli. J. Basic. Microbiol..

[52] Jha Y., Mohamed H.I. (2023). Inoculation with Lysinibacillus Fusiformis Strain YJ4 and Lysinibacillus Sphaericus Strain YJ5 Alleviates the Effects of Cold Stress in Maize Plants. Gesunde Pflanz..

[53] Hashmi I., Bindschedler S., Junier P. (2020). Firmicutes. Beneficial Microbes in Agro-Ecology.

[54] Thatoi H., Mishra R.R., Behera B.C. (2020). Biotechnological Potentials of Halotolerant and Halophilic Bacteria from Mangrove Ecosystems. Biotechnological Utilization of Mangrove Resources.

[55] Bouteau H.E.-M., Job C., Job D., Corbineau F., Bailly C. (2007). ROS Signaling in Seed Dormancy Alleviation. Plant Signal. Behav..

[56] El-Maarouf-Bouteau H., Sajjad Y., Bazin J., Langlade N., Cristescu S.M., Balzergue S., Baudouin E., Bailly C. (2015). Reactive Oxygen Species, Abscisic Acid and Ethylene Interact to Regulate Sunflower Seed Germination: ROS, Ethylene and ABA in Seed Germination. Plant Cell Environ..

[57] Saini P., Gani M., Kaur J.J., Godara L.C., Singh C., Chauhan S.S., Francies R.M., Bhardwaj A., Bharat Kumar N., Ghosh M.K., Zargar S.M., Zargar M.Y. (2018). Reactive Oxygen Species (ROS): A Way to Stress Survival in Plants. Abiotic Stress-Mediated Sensing and Signaling in Plants: An Omics Perspective.

[58] Fouts D.E., Tyler H.L., DeBoy R.T., Daugherty S., Ren Q., Badger J.H., Durkin A.S., Huot H., Shrivastava S., Kothari S. (2008). Complete Genome Sequence of the N2-Fixing Broad Host Range Endophyte Klebsiella Pneumoniae 342 and Virulence Predictions Verified in Mice. PLoS Genet..

[59] Rajkumari J., Paikhomba Singha L., Pandey P. (2018). Genomic Insights of Aromatic Hydrocarbon Degrading Klebsiella Pneumoniae AWD5 with Plant Growth Promoting Attributes: A Paradigm of Soil Isolate with Elements of Biodegradation. 3 Biotech..

[60] Shuai H., Meng Y., Luo X., Chen F., Zhou W., Dai Y., Qi Y., Du J., Yang F., Liu J. (2017). Exogenous Auxin Represses Soybean Seed Germination through Decreasing the Gibberellin/Abscisic Acid (GA/ABA) Ratio. Sci. Rep..

[61] Rueda-Puente E.O., García-Hernández J.L., Preciado-Rangel P., Murillo-Amador B., Tarazón-Herrera M.A., Flores-Hernández A., Holguin-Peña J., Aybar A.N., Barrón Hoyos J.M., Weimers D. (2007). Germination of *Salicornia bigelovii* Ecotypes under Stressing Conditions of Temperature and Salinity and Ameliorative Effects of Plant Growth-promoting Bacteria. J. Agron. Crop Sci..

[62] Fiodor A., Ajijah N., Dziewit L., Pranaw K. (2023). Biopriming of Seed with Plant Growth-Promoting Bacteria for Improved Germination and Seedling Growth. Front. Microbiol..

[63] Kumar V., Sharma N., Maitra S.S., Lakkaboyana S.K. (2020). In Vivo Removal of Profenofos in Agricultural Soil and Plant Growth Promoting Activity on *Vigna radiata* by Efficient Bacterial Formulation. Int. J. Phytoremediation.

[64] Abdelaal K., AlKahtani M., Attia K., Hafez Y., Király L., Künstler A. (2021). The Role of Plant Growth-Promoting Bacteria in Alleviating the Adverse Effects of Drought on Plants. Biology.

[65] Bektaş İ., Küsek M. (2021). The Isolation and Characterization of Phosphate Solubilizing Bacteria from the Onion Rhizosphere and Their Effect on Onion Growth. Kahramanmaraş Sütçü İmam Üniversitesi Tarım Ve Doğa Derg..

[66] Bahadir P.S., Liaqat F., Eltem R. (2018). Plant Growth Promoting Properties of Phosphate Solubilizing Bacillus Species Isolated from the Aegean Region of Turkey. Turk. J. Bot..

[67] Mohamed E.A.H., Farag A.G., Youssef S.A. (2018). Phosphate Solubilization by *Bacillus subtilis* and *Serratia marcescens* Isolated from Tomato Plant Rhizosphere. JEP.

[68] Ahmad I., Ahmad M., Hussain A., Jamil M. (2021). Integrated Use of Phosphate-Solubilizing *Bacillus subtilis* Strain IA6 and Zinc-Solubilizing Bacillus Sp. Strain IA16: A Promising Approach for Improving Cotton Growth. Folia Microbiol..

