# Supplementary material for: The Impact of Rhizospheric and Endophytic Bacteria on the Germination of Carajasia cangae: A Threatened Rubiaceae of the Amazon Cangas
Source: Microorganisms. 2024 Sep 6;12(9):1843. doi: 10.3390/microorganisms12091843 (PMC11433783; doi:10.3390/microorganisms12091843)
Supplement: Supplementary file 1 [file microorganisms-12-01843-s001.zip › microorganisms-3159115-supplementary.pdf]

Impact of rhizospheric and endophytic bacteria on the germination of *Carajasia cangae*: a threatened Rubiaceae of Amazon cangas

Daniela Boanares [1], Aline Figueiredo Cardoso [1], Diego Fernando Escobar Escobar [1], Keila Jamille Alves Costa [1], José Augusto Bitencourt [1], Paulo Henrique O. Costa [1], Silvio Ramos [1], Markus Gastauer [1], Cecilio Frois Caldeira [1,*]

Instituto Tecnológico Vale, Belém 66055-090, PA, Brazil.
* Correspondence: cecilio.caldeira@itv.org

Supplemental Table S1. Most abundant bacterial taxa identified in soils associated with *Carajasia cangae*.

	Identification source	Identified Phyla (Relative Abundance %)	Identified Genera (Relative Abundance %)
**BACTERIA**	*C1*	*Proteobacteria (45.5); Actinobacteria (29.8); Acidobacteria (19.6); Bacteroidetes (3.75); Chloroflexi (1.53)*	*Unidentified (41.9); Mycobacterium (25.5); Acidothermus (12.0); Acidibacter (7.76); Bryobacter (7.75); Mucilaginibacter (51.1)*
	C2	*Proteobacteria (47.3); Actinobacteria (29.4); Acidobacteria (17.8); Bacteroidetes (3.66); Chloroflexi (1.85)*	*Unidentified (42.9); Mycobacterium (20.3); Acidothermus (11.3); Acidibacter (6.67); Caballeronia (6.34); Conexibacter (4.46)*
	C3	*Proteobacteria (52.7); Actinobacteria (21.4); Acidobacteria (19.6); Bacteroidetes (4.92); Chloroflexi (1.44)*	*Bryobacter (26.0); Ralstonia (25.3); Mycobacterium (12.2); Asticcacaulis (10.2); Streptomyces (7.2); Mucilaginibacter (7.0)*
	C4	*Actinobacteria (62.2); Proteobacteria (24.6); Acidobacteria (12.1); Bacteroidetes (1.14)*	*Mycobacterium (42.1); Acidothermus (23.8); Uncultured (18.7); Caballeronia (6.79); Conexibacter (4.89); Occallatibacter (3.77)*
**BACTERIA**	*C5*	*Actinobacteria (57.0); Proteobacteria (28.3); Acidobacteria (13.2); Bacteroidetes (1.53)*	*Mycobacterium (36.4); Acidothermus (23.8); uncultured (15.8); Caballeronia (11.6); Occallatibacter (7.2); Conexibacter (5.25)*
	C6	*Proteobacteria (42.4); Actinobacteria (39.7); Acidobacteria (15.5); Bacteroidetes (2.31)*	*Mycobacterium (31.3); Uncultured (16.6) Bryobacter (13.1); Acidothermus (11.9); Caballeronia (10.7); Bradyrhizobium (4.72)*
	C7	*Actinobacteria (44.2); Proteobacteria (29.0); Acidobacteria (26.8)*	*Mycobacterium (38.9); Bryobacter (30.1); Acidothermus (11.2); uncultured (8.37); Pajaroellobacter (4.16); Crossiella (3.64)*
	C8	*Actinobacteria (56.7); Proteobacteria (31.7); Acidobacteria (7.19); Bacteroidetes (4.39)*	*Mycobacterium (50.9); uncultured (14.2); Acidothermus (11.5); Streptomyces (5.88); Mucilaginibacter (5.87); Crossiella (4.23)*
	C9	*Proteobacteria (34.5); Acidobacteria (27.3); Actinobacteria (25.5); Bacteroidetes (6.96); Firmicutes (5.74)*	*Mycobacterium (28.0); Bryobacter (23.4); Mucilaginibacter (10.1); uncultured (9.61); Fonticella (7.48); Acidothermus (6.33)*

Supplemental Table S2: Most abundant bacterial taxa identified and bacterial 16S rRNA sequences in different samples associated with *Carajasia cangae* growing in a canga in Serra dos Carajás, Eastern Amazon.

**OTU***	**Abundance**	**Sample**	**Phylum**	**Class**	**Order**	**Family**	**Genus**	**Species**
OTU695897805	0.657895	E1	Firmicutes	Bacilli	Bacillales	Bacillaceae	Bacillus	Bacillus cereus
OTU755204678	0.342105	E1	Firmicutes	Bacilli	Bacillales	Bacillaceae	Bacillus	Bacillus subtilis
OTU695897805	0.975	E2	Firmicutes	Bacilli	Bacillales	Bacillaceae	Bacillus	Bacillus cereus
OTU248802708	0.025	E2	Proteobacteria	Gammaproteobacteria	Enterobacteriales	Enterobacteriaceae	Klebsiella	Klebsiella pneumoniae
OTU248802708	1	E3	Proteobacteria	Gammaproteobacteria	Enterobacteriales	Enterobacteriaceae	Klebsiella	Klebsiella pneumoniae
OTU695897805	1	E4	Firmicutes	Bacilli	Bacillales	Bacillaceae	Bacillus	Bacillus cereus
OTU695897805	0.909091	E5	Firmicutes	Bacilli	Bacillales	Bacillaceae	Bacillus	Bacillus cereus
OTU693716741	0.090909	E5	Firmicutes	Bacilli	Bacillales	Planococcaceae	Lysinibacillus	Lysinibacillus fusiformis
OTU695897805	1	E6	Firmicutes	Bacilli	Bacillales	Bacillaceae	Bacillus	Bacillus cereus
OTU695897805	0.71	E7	Firmicutes	Bacilli	Bacillales	Bacillaceae	Bacillus	Bacillus cereus
OTU755204678	0.29	E7	Firmicutes	Bacilli	Bacillales	Bacillaceae	Bacillus	Bacillus subtilis
OTU695897805	1	R1	Firmicutes	Bacilli	Bacillales	Bacillaceae	Bacillus	Bacillus cereus
OTU695897805	0.666667	R2	Firmicutes	Bacilli	Bacillales	Bacillaceae	Bacillus	Bacillus cereus
OTU166536478	0.333333	R2	Firmicutes	Bacilli	Bacillales	Planococcaceae	Planomicrobium	uncultured bacterium
OTU693716741	1	R3	Firmicutes	Bacilli	Bacillales	Planococcaceae	Lysinibacillus	Lysinibacillus fusiformis
OTU695897805	0.00301	R4	Firmicutes	Bacilli	Bacillales	Bacillaceae	Bacillus	Bacillus cereus
OTU436687366	0.984748	R4	Proteobacteria	Gammaproteobacteria	Enterobacteriales	Enterobacteriaceae	Serratia	uncultured bacterium
OTU894036020	0.007827	R4	Proteobacteria	Gammaproteobacteria	Enterobacteriales	Enterobacteriaceae	Serratia	Serratia marcescens
OTU908196646	0.004415	R4	Proteobacteria	Gammaproteobacteria	Enterobacteriales	Enterobacteriaceae	Serratia	Serratia sp. R46
OTU695897805	0.002209	R5	Firmicutes	Bacilli	Bacillales	Bacillaceae	Bacillus	Bacillus cereus
OTU436687366	0.984704	R5	Proteobacteria	Gammaproteobacteria	Enterobacteriales	Enterobacteriaceae	Serratia	uncultured bacterium
OTU894036020	0.007648	R5	Proteobacteria	Gammaproteobacteria	Enterobacteriales	Enterobacteriaceae	Serratia	Serratia marcescens
OTU908196646	0.005099	R5	Proteobacteria	Gammaproteobacteria	Enterobacteriales	Enterobacteriaceae	Serratia	Serratia sp. R46
OTU890204875	0.00034	R5	Proteobacteria	Gammaproteobacteria	Enterobacteriales	Enterobacteriaceae	Serratia	Serratia sp. R36

OTU695897805	1	R6	Firmicutes	Bacilli	Bacillales	Bacillaceae	Bacillus	Bacillus cereus
OTU695897805	0.999988	R7	Firmicutes	Bacilli	Bacillales	Bacillaceae	Bacillus	Bacillus cereus
OTU755204678	5.85E+08	R7	Firmicutes	Bacilli	Bacillales	Bacillaceae	Bacillus	Bacillus subtilis
OTU436687366	5.85E+08	R7	Proteobacteria	Gammaproteobacteria	Enterobacteriales	Enterobacteriaceae	Serratia	uncultured bacterium
OTU695897805	0.875	R8	Firmicutes	Bacilli	Bacillales	Bacillaceae	Bacillus	Bacillus cereus
OTU166536478	0.0625	R8	Firmicutes	Bacilli	Bacillales	Planococcaceae	Planomicrobium	uncultured bacterium
OTU693716741	0.0625	R8	Firmicutes	Bacilli	Bacillales	Planococcaceae	Lysinibacillus	Lysinibacillus fusiformis
OTU695897805	1	R9	Firmicutes	Bacilli	Bacillales	Bacillaceae	Bacillus	Bacillus cereus

* Operational Taxonomic Units
